# Protective Effects of Socioeconomic Status and Lifestyle on Amyloid‐ and White Matter Hyperintensity‐Related Longitudinal Brain Atrophy and Cognitive Decline

**DOI:** 10.1002/ana.70022

**Published:** 2025-08-09

**Authors:** Dario Bachmann, Maha Wybitul, Sandro Studer, Antje Saake, Katrin Rauen, Andreas Buchmann, Bettina von Rickenbach, Esmeralda Gruber, Roger M. Nitsch, Christoph Hock, Anton Gietl, Valerie Treyer

**Affiliations:** ^1^ Institute for Regenerative Medicine University of Zurich Zurich Switzerland; ^2^ Neuroscience Center Zurich University of Zurich Zurich Switzerland; ^3^ Clinic for Aging Medicine, Hospital Affoltern Affoltern Switzerland; ^4^ Neurimmune AG Zurich Switzerland; ^5^ Center for Prevention and Dementia Therapy University of Zurich Zurich Switzerland; ^6^ Department of Geriatric Psychiatry Psychiatric Hospital Zurich Zurich Switzerland; ^7^ Department of Nuclear Medicine University Hospital of Zurich, University of Zurich Zurich Switzerland

## Abstract

**Objective:**

Socioeconomic status (SES) and lifestyle activities (LA) are strongly related, and both are associated with dementia risk. We investigated the influence of SES and LA on brain atrophy and cognitive decline considering amyloid‐beta (Aβ) positron emission tomography and white matter hyperintensity (WMH) load.

**Methods:**

We investigated 221 older adults (mean age, 67.1 ± 8.2 years) who underwent cognitive testing annually over a median follow‐up period of 3.4 years. Longitudinal T1‐weighted magnetic resonance imaging was available for 181 participants. Measures of SES and LA were collected using questionnaires and combined to SES and LA scores using a latent variable approach. We used linear mixed‐effects models to investigate if SES and LA are independently or interactively with Aβ and WMH load associated with decline in domains language, processing speed/attention, executive function, and episodic memory. Mediation models assessed whether these associations were explained by gray matter atrophy.

**Results:**

SES and LA were associated with better cognitive performance at baseline, but were not associated with cognitive decline over time. In interaction with brain pathologies, higher LA reduced the detrimental effects of Aβ and WMH load on language decline, whereas higher SES reduced the detrimental effects of Aβ pathology on episodic memory decline. Individuals with low SES showed faster Aβ‐related gray matter atrophy, which mediated the association between Aβ and episodic memory decline but not language decline.

**Interpretation:**

These results suggest that multiple resilience mechanisms underlie the protective effects of SES and LA on cognitive decline. Interventions targeting SES‐related risk factors may be most effective when implemented early, before neurodegenerative changes begin. ANN NEUROL 2025;98:1222–1236

Cerebrovascular disease and Alzheimer's disease (AD) pathology are 2 major causes of cognitive decline in the elderly.^1^ Imaging markers of these pathologies such as white matter hyperintensities (WMH) and amyloid‐beta (Aβ) deposits are known to predict cognitive deterioration, but the rate of cognitive decline can vary greatly between individuals.^2^, ^3^, ^4^ Previous research has highlighted the role of socioeconomic status (SES) and lifestyle activities (LA) such as social, physical, and cognitive activities as likely candidates that influence cognitive trajectories.^5^ However, it is unclear whether SES and LA influence cognitive decline by affecting WMH and Aβ burden or if they instead mitigate the adverse downstream effects of these pathologies on brain atrophy and cognition.

SES is a complex concept that defines an individual's standing in society based on a combination of factors including income, education, and occupation.^6^ Higher SES is associated with better health, which is reflected in studies reporting a lower incidence of various disorders, including dementia, in individuals of high SES.^7^, ^8^ SES and LA are strongly related,^9^, ^10^ and the highest dementia risk has been found for individuals of low SES with unhealthy lifestyles.^11^ However, despite mounting evidence for the protective role of SES and LA, the mechanisms through which they operate remain insufficiently understood.^5^, ^12^, ^13^, ^14^ SES and LA may protect against the development of WMHs and Aβ pathology, for instance, by reducing exposure to risk factors.^5^ Alternatively, they may increase resilience to these pathologies by supporting cognitive reserve, brain maintenance, and brain reserve.^15^, ^16^ These concepts have been proposed to help explain disparities in cognitive trajectories among individuals with similar levels of brain pathology. Cognitive reserve refers to the brain's ability to sustain cognitive performance that is better than expected given the degree of aging‐ or pathology‐related brain changes. Brain maintenance refers to the relative absence of brain changes over time, such as reduced brain atrophy. While brain maintenance concerns the rate of change, brain reserve is considered a static construct that involves, for instance, a higher brain volume at a given point of time. Hence, brain reserve refers to the brain's structural resources that provide a buffer against damage.

Knowledge of how and under what circumstances SES and LA influence the risk of cognitive decline is important for developing more effective, personalized interventions to help reduce the dementia burden. This knowledge could also facilitate the identification of subgroups at high risk of disease progression, which should undergo increased monitoring or may be most suitable for disease‐modifying treatments. Given the interrelation between SES and LA, it is crucial to consider both factors simultaneously to determine whether they independently contribute to cognitive resilience and whether distinct processes underlie their effects. Therefore, in the present study, we investigated if SES and LA influence the rate of cognitive decline in a cohort of older adults without dementia. We first examined whether these factors are associated with domain‐specific cognitive decline independently of, or in interaction with, Aβ or WMH burden. We, then, assessed whether the observed associations are mediated by longitudinal gray matter atrophy.

## Methods

### Study Population

Participants are enrolled in the IDcog study,^17^ a longitudinal, community‐based cohort study of cognitive decline conducted at the Center for Prevention and Dementia Therapy at the Institute for Regenerative Medicine, University of Zurich, Switzerland. Participants were recruited with local advertising. For enrollment into the IDcog study, participants had to be at least 50 years of age and German‐speaking. Individuals with comorbid conditions that could interfere with baseline or follow‐up cognitive assessments, or magnetic resonance imaging (MRI) and positron emission tomography (PET) procedures were excluded. Participants are followed longitudinally with detailed neuropsychological testing and clinical evaluation in an approximately annual timespan. Based on the cognitive performance and functional assessment in the clinical evaluation, participants were categorized as cognitively unimpaired (CU) or having mild cognitive impairment (MCI) in accordance with published diagnostic guidelines.^18^ For the present analysis, 7 participants were excluded because of missing follow up neuropsychological assessments and 5 participants were excluded because all LA variables were missing, leaving 221 participants with Aβ PET and longitudinal neuropsychological data. Of these, baseline and follow‐up T1‐weighted MRI scans were available for 182 participants of which we excluded 1 participant because of insufficient T1‐weighted image quality, leaving 181 participants included in the gray matter atrophy analysis. Images were generally acquired within a few days of the baseline and last neuropsychological assessments. The study was approved by the ethics committee of the Canton Zurich. All participants gave written informed consent.

### Imaging Acquisition

T1‐weighted MRI scans were acquired on a 3 T 750 W or Premier scanner model, equipped with either a 32‐channel or 48‐channel coil scanner (GE Healthcare, Waukesha, WI). A high‐resolution 3D T1‐weighted fast spoiled gradient recalled (FSPGR) sequence was acquired, with an isotropic voxel size of 0.5mm, sagittal slice orientation, and repetition time (TR) = 11ms, echo time (TE) = 5.2ms, and inversion time (TI) = 600ms, and a flip angle of 8°. Fluid‐attenuated inversion recovery (FLAIR) and PET imaging was performed on a 3 T Signa PET/MR scanner (GE Healthcare). High resolution cube FLAIR images (0.48 × 0.48 × 0.6mm voxel size) were acquired with TR/TE/TI = 6,502/130.7/1,962ms, and flip angle of 90°.

[18F]‐flutemetamol PET images were acquired from 80 to 110 minutes after injection of approximately 140MBq [18F]‐flutemetamol. A BRAVO 3D T1‐weighted image with isotropic voxel size of 1mm was acquired in parallel to the PET acquisition for brain segmentation.

### Image Processing

T1‐weighted images first underwent quality assessment using the CAT12 toolbox, during which the total intracranial volume (TIV) was also extracted. For longitudinal voxel‐wise analyses, baseline and follow‐up T1‐weighted images for each patient were processed using SPM12's pairwise longitudinal registration, generating a midpoint average T1‐weighted image and a 3‐dimensional (3D) Jacobian rate map, which reflects the annualized rate of volumetric change. To enhance inter‐subject alignment, a sample‐specific template was created from the midpoint images using the Diffeomorphic Anatomical Registration Through Exponentiated Lie Algebra (DARTEL) toolbox.^19^ Individual midpoint images were then registered to this sample‐specific template, and subsequently normalized to Montreal Neurological Institute space using the DARTEL flow fields. Finally, the normalized Jacobian rate maps were smoothed with an 8mm full‐width at half‐maximum (FWHM) Gaussian kernel. Smoothed and normalized Jacobian rate maps were then used for voxel‐based morphometry analyses using the VoxelStats package (v1.2; github.com/sulantha2006/VoxelStats) for MATLAB (The MathWorks, Natick, MA).^20^

White matter lesions were segmented by the lesion prediction algorithm as implemented in the LST toolbox (www.statistical-modelling.de/lst.html) for statistical parametric mapping. Lesion probability maps were binarized using a threshold of 0.65.^21^ Lesion masks were then visually inspected and manually corrected if necessary. Global WMH volume was quantified and adjusted for TIV by calculating the global WMH volume percentage of the TIV. WMH volume was unavailable for 9 participants because of artifacts on the FLAIR images. For the visualization of the results, we designated individuals with a normalized WMH volume in the highest tertile as having an increased WMH burden.

[18F]‐flutemetamol PET image analysis was performed using PMOD NeuroTool (version 3.9, PMOD Technologies). A global Aβ standardized uptake value ratio (SUVR) was determined on the averaged frames from 85 to 105 minutes post‐injection in a global composite region that included left and right frontal, temporal, and parietal cortices and posterior and anterior cingulate gyrus normalized to the uptake in the cerebellar gray matter. For the visualization of the results and the mediation analysis, we applied a threshold of 12 Centiloids (CL) to classify individuals as Aβ+ (CL ≥12) or Aβ− (CL <12).

### Neuropsychological Assessment

Cognitive performance was assessed approximately annually. We created 4 composite scores summarizing the domains of language function, processing speed/attention, executive function, and episodic memory. Language included the animal fluency task (total after 3 minutes) and the 15‐word version of the Boston Naming Test. Processing speed/attention included Stroop I (word), Trail Making Test (TMT) part A, Stroop III (color‐word), and the digit span forward. Executive function included s‐words (correct new words in third minute), digit span backward, and TMT part B. Episodic memory included immediate recall, delayed recall (words and figures), and recognition from the Consortium to Establish a Registry for Alzheimer's Disease battery. The mean and standard deviation of the cognitively unimpaired sub‐sample were used to calculate z‐scores for all time points. Where necessary, we reversed the z‐scores of tests so that a higher score represents better performance across all tests.

### SES and Lifestyle‐Related Variables

SES and lifestyle data were collected at baseline for all participants. The primary SES and LA variables in this study included years of education, net income, current cognitive activity (CA), current physical activity (PA), early‐ and mid‐life activities, educational complexity, and occupation.

Years of education referred to the total number of years of formal schooling (range: 9–20 years). Net income was reported as the current available monthly income, categorized into 6 levels, from less than 1,000 Swiss francs (level 1) to more than 7,000 Swiss francs (level 6). Current CA and PA were assessed via a questionnaire covering participants' activities over the past 12 months.^22^ The current CA score was based on 11 items that measured the frequency of tasks such as reading, solving puzzles, social activities, and other cognitively demanding activities, with scores ranging from 0 to 70. The current PA score was based on 6 items focusing on activities such as walking, sports or exercise, with total scores ranging from 0 to 42.

Data on educational complexity, occupation, and early life and mid‐life activities were collected using the Lifetime of Experiences Questionnaire,^23^ which covers 3 life stages: early life (ages 13–30), mid‐life (ages 30–65), and late life (ages 65+). Educational complexity in early life was assessed through specific questions about participants' educational experiences during ages 13 to 30, reflecting both the extent and nature of these experiences. Occupation in mid‐life (ages 30–65) was evaluated through specific questions about participants' professional history, with occupations classified into 9 categories. Management responsibilities were also considered by asking how many employees or colleagues participants supervised. In both the early life and mid‐life stages, non‐specific activities were assessed, encompassing a wide range of experiences such as playing musical instruments, drawing, reading, socializing, visiting family, participating in sports, learning new languages, traveling, and pursuing hobbies. These activities were evaluated for frequency, with scores ranging from 0 to 35. Late life activities were not included in the analysis, as many participants had not yet reached the age of 65. Details regarding score calculations can be found in the original publication.^23^

### Confounder Assessment

Potential confounders were assessed at clinical visits during the patient anamnesis or through questionnaires. Lifestyle‐related confounders included smoking status (ever smoker vs never smoker), alcohol consumption (user vs non‐user), and living situation (living alone vs living with others). We systematically assessed comorbidity burden using the cumulative illness rating scale (CIRS),^24^ which rates the burden of chronic medical illness across 14 bodily systems while considering the severity of the condition. Cardiovascular disease risk was assessed using the lipid‐based Framingham Heart Study cardiovascular disease risk score (FHS‐CVD),^25^ which provides a 10‐year probability of future cardiovascular events.

### Establishment of SES and LA Variables Using Confirmatory Factor Analysis

Because SES and LA are composed of multiple related factors rather than single observable variables, we modeled both as latent variables to capture their underlying shared variance. This approach allowed us to estimate SES and LA even when some indicators were missing, ensuring all participants received a score.

**FIGURE 1:**
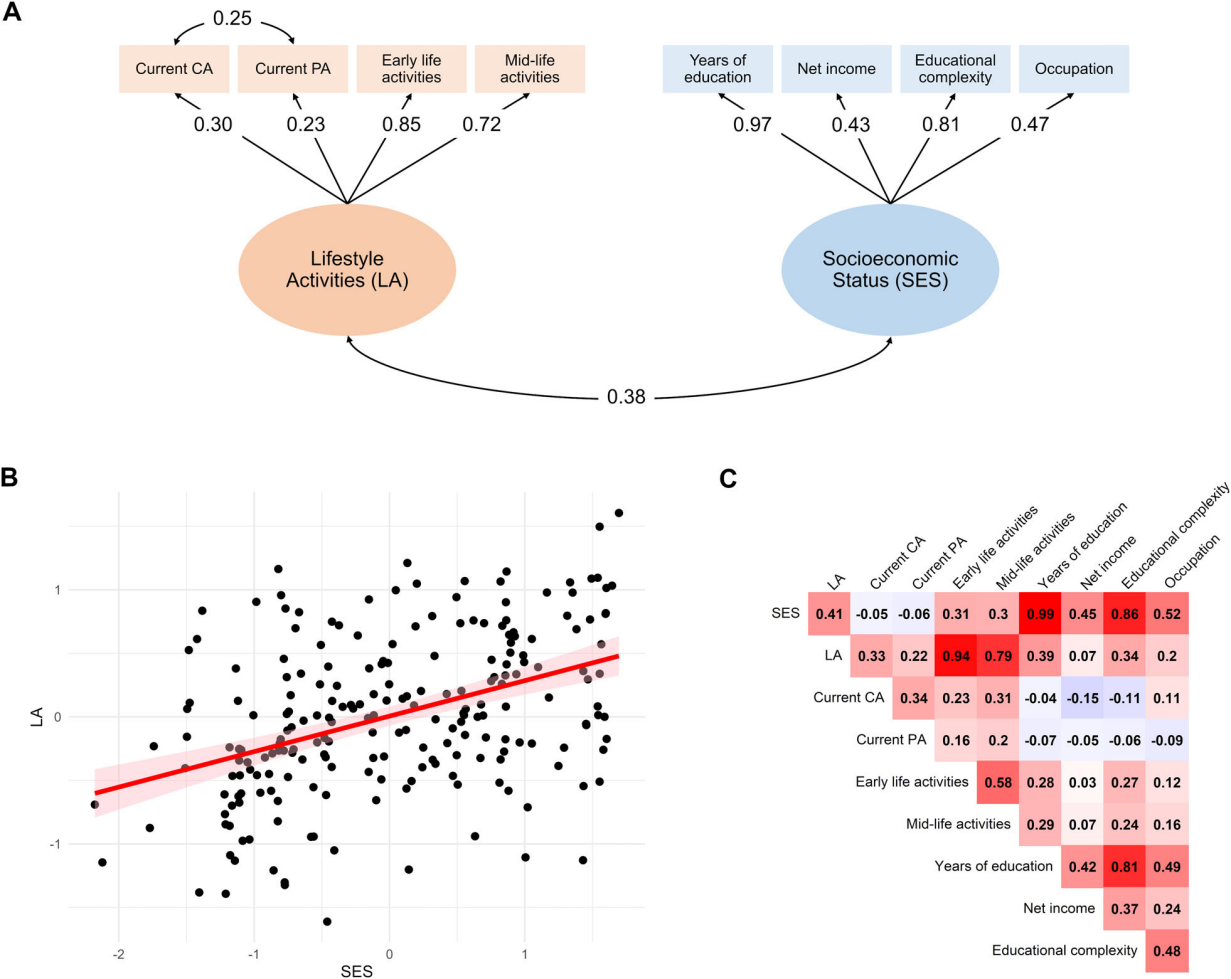
Associations among lifestyle activities (LA) and socioeconomic status (SES) variables. A shows the latent variables for LA and SES from which factor scores were derived. B shows the correlation between the extracted factor scores. C presents a correlation matrix of Spearman's ρ that shows the correlation between the factor scores for LA and SES and all the individual indicators used to derive these scores. [Color figure can be viewed at www.annalsofneurology.org]

Each latent variable was inferred from 4 observed indicators. For SES, we used years of education, net income, educational complexity, and occupation, whereas for LA, we used current CA, current PA, early life activities, and mid‐life activities. A 2‐factor confirmatory factor analysis (CFA) was conducted using the *cfa* function from the lavaan package. The initial model fit was suboptimal (Comparative Fit Index (CFI) = 0.92, Tucker‐Lewis Index (TLI) = 0.88, Root Mean Square Error of Approximation (RMSEA) = 0.10, Standardized Root Mean Square Residual (SRMR) = 0.07). Modification indices suggested that allowing correlations between the error terms (ie, the portions of the observed variables not explained by the latent variables) for current CA and PA would improve the model fit. Making this modification is justified, as both scores were obtained using similar questionnaires and reflect current activity levels. Including this correlation improved the model fit (CFI = 0.95, TLI = 0.92, RMSEA = 0.08, SRMR = 0.06). Further modifications suggested by modification indices, such as correlating errors between current CA and education, were avoided to prevent overfitting and to maintain conceptual distinctions between the latent constructs. The 2 latent constructs used to estimate SES and LA are shown in Figure 1A. Factor scores were then predicted using a least squares regression approach.^26^ We also tested an alternative model in which all indicators loaded onto a single latent variable. However, this model demonstrated poor fit to the data (CFI = 0.74, TLI = 0.62, RMSEA = 0.17, SRMR = 0.11), supporting the distinctiveness of the LA and SES latent variables.

### Statistical Analysis

Statistical analysis was conducted in R version 4.4.1. Spearman's ρ was used to examine the correlations among the SES and LA indicators. Two multivariat linear regression models were conducted to estimate the relationship between SES and LA with Aβ burden, WMH volume, age, sex, APOE4 carrier status, and baseline diagnosis (CU vs MCI). All variables were standardized.

As a first step, we investigated whether SES and LA, independently or in interaction with Aβ burden and WMH volume, were associated with cognitive decline. We used linear mixed‐effects models to examine change in cognitive performance, accounting for both fixed and random effects. Random intercepts and slopes were specified to capture individual variation in baseline cognition and cognitive trajectories over time. Models were fitted with the nlme package. Each model included the covariates baseline age, sex, APOE4 carrier status, and all 2‐way interactions of these variables with time (years since baseline). For each cognitive domain —language, processing speed/attention, executive function, and episodic memory—we ran the following 3 linear mixed‐effects models:

**Model A:** *Cognition* ~ *Score*_LA_ _/SES_ × *time *+ Aβ × *time *+ WMH × *time *+ *age *× *time *+ *sex *× *time* + APOE4 × *time *+ *Score*_LA/SES_ + Aβ + WMH + *age* + *sex* + APOE4 + *time *+ (1 + *time* | *participant*).

**Model B:** *Cognition* ~ *Score*_LA/SES_ × Aβ × *time* + *age* × *time* + *sex* × *time* + APOE4 × *time* + *Score*_LA/SES_ × Aβ + *Score*_LA/SES_ × *time* + Aβ × *time* + *Score*_LA/SES_ + Aβ + *age* + *sex* + APOE4 + *time* + (1 + *time*|*participant*).

**Model C:** *Cognition* ~ *Score*_LA/SES_ × WMH × *time* + *age* × *time*+ *sex* × *time* + APOE4 × *time* + *Score*_LA/SES_ × WMH + *Score*_LA/SES_ × *time* + WMH × *time* + *Score*_LA/SES_ + WMH + *age* + *sex* + APOE4 + *time* + (1 + *time* | *participant*).

where cognition represents the composite scores for each cognitive domain, and Score_LA/SES_ represents either LA or SES scores extracted from the latent variables. All continuous variables, except for time, were standardized before model fitting to facilitate comparison of effect sizes. The primary focus was on the 3‐way interactions in models B and C (ie, *Score*_LA/SES_ × Aβ × *time* and *Score*_LA/SES_ × WMH × *time*), which tested whether LA or SES moderated the relationship between baseline levels of Aβ or WMH and cognitive change. A significant 3‐way interaction involving time suggests a synergistic effect whereby LA or SES with Aβ or WMH burden influence cognitive change beyond their separable effects. The main effect of *Score*_LA/SES_ represents its association with cognition at baseline (at time = 0). If both 3‐way interactions (ie, *Score*_LA/SES_ × Aβ × *time* and *Score*_LA/SES_ × WMH × *time*) were significantly associated with cognitive change in the same cognitive domain, both interactions were included in the same model to assess whether these associations were independent of each other. False discovery rate (FDR) was corrected for 8 comparisons per *Score*_LA/SES_. All models were subsequently repeated with adjustments for confounders. In secondary analyses, we explored the interactions in samples stratified by sex and APOE4 carrier status. In sensitivity analyses, we repeated the primary analysis after excluding the current CA and PA indicators from the LA latent construct, therefore, deriving the LA score solely from early‐ and mid‐life activities. Additionally, we excluded the early‐ and mid‐life activity indicators, therefore, deriving the LA score solely from current activities. These analyses were intended to explore the likelihood that our findings were influenced by reverse causality. We also repeated the analysis after excluding the years of education indicator from the SES latent construct, as its factor loading indicates that it is nearly identical to the latent variable, which may reduce the contribution of other indicators. Finally, we repeated the analysis after excluding APOE4 as a covariate as it shows strong associations with Aβ‐PET burden.^17^

Given that LA potentially mediates the relationship between SES and cognitive decline,^27^, ^28^ we used moderated mediation to explore whether SES affects cognitive decline directly or indirectly through its effect on LA, and whether this effect is moderated by Aβ or WMH burden. Moderated mediation analyses were conducted only when both LA and SES significantly moderated the relationship between Aβ or WMH load and cognitive decline in the linear mixed‐effects models. Individual slopes of cognitive decline were estimated and extracted from linear mixed‐effects models with random intercepts and slopes per participant and time as the sole predictor. We also present the results of a final multigroup model, which provides more accurate estimates by separately estimating only those paths where significant group differences were observed, while estimating the remaining paths as a single parameter. Significant differences between groups for each path were tested using likelihood ratio tests. Models were adjusted for age, sex, and APOE4. The moderated mediation models were implemented in the lavaan package with 1,000 bootstrap samples to estimate confidence intervals and a path was deemed significant if the 95% confidence interval did not include zero.

As a second step, we examined whether SES and LA influence cognitive decline by moderating the associations between Aβ or WMH burden, brain atrophy, and cognitive decline. Voxel‐wise linear regression analyses, adjusted for age, sex, and TIV, were used to investigate whether Aβ burden, WMH burden, SES, and LA predict longitudinal gray matter atrophy, using separate models for each predictor. The mean rate of gray matter volume change was then extracted from regions that survived random field theory correction with *p* < 0.001 at voxel level and cluster threshold set at *p* < 0.05.^20^ We used a moderated mediation model to examine whether the extracted gray matter atrophy rate mediates the association between Aβ SUVR and domain‐specific cognitive slopes, and whether this effect is moderated by SES or LA. SES and LA were binarized using a median split. Given that men showed significantly higher SES compared to women, we also examined the moderated mediation model within subsamples of men and women.

## Results

### Cohort Characteristics

The total cohort for the present analysis included 221 participants of which 181 participants had longitudinal T1‐weighted MRI data. Median follow up was 3.4 years (interquartile range: 3.1–4.1). At baseline, participants had a mean age of 67 ± 8.2 years and 104 (47.1%) were female. Of the 48 (21.7%) participants who had MCI, 19 (39.6%) had a CL >12 indicating elevated Aβ pathology. Cohort characteristics are summarized in Table 1. Cohort characteristics of the sample with longitudinal T1‐weighted MRI are summarized in Table S1.

**TABLE 1:** Cohort Characteristics

	Participants (n = 221)
Age at baseline, mean yr (SD) [range]	67.1 (8.2) [51–90]
Sex, F, n (%)	104 (47.1)
MMSE, mean (SD)	29.2 (1.1)
MCI, n (%)	48 (21.7)
APOE4 carriers, n (%)	52 (23.5)
Aβ‐PET burden, mean SUVR (SD)	1.27 (0.22)
Centiloid >12, n (%)	72 (32.6)
WMH volume, median % of ICV (IQR), (NA = 9)	0.17 [0.08–0.36]
No. of NP assessments, 2/3/4/5, n	43/56/95/27
NP Follow‐up yr, median yr (IQR)	3.4 (3.1–4.1)
Follow up T1 MRI, n (%)	181 (81.9)
T1 MRI Follow‐up yr, median yr (IQR)	3.4 (3.1–4.3)
Baseline total CIRS score, median (IQR)	6 [4–9]
Baseline FHS‐CVD risk score (in %), mean (SD)	18.4 (0.13)
Alcohol use, n (%)	42 (19.9)
Smoker (past or present), n (%)	114 (51.6)
SES indicators	
Yr of education, mean (SD)	15.4 (2.9)
Net income, mean (SD), (NA = 31)	4.7 (1.3)
Educational complexity score, mean (SD), (NA = 2)	11.9 (5.8)
Occupation score, mean (SD), (NA = 2)	11.4 (2.6)
LA indicators	
Current CA, mean (SD), (NA = 1)	23.3 (7.2)
Current PA, mean (SD), (NA = 1)	13.4 (6)
Early life activities score, mean (SD)	23.3 (4.5)
Mid‐life activities score, mean (SD)	23.3 (4.6)

Aβ = amyloid‐beta; APOE4 = apolipoprotein E4; CA = cognitive activity; CIRS = cumulative illness rating scale; FHS‐CVD = Framingham Heart Study cardiovascular disease; IQR = interquartile range; LA = lifestyle activities; MCI = mild cognitive impairment; MMSE = Mini‐Mental State Examination; NA = not available; NP = neuropsychological; PA = physical activity; PET = positron emission tomography; SES = socioeconomic status; SD = standard deviation; SUVR = standardized uptake value ratio; WMH = white matter hyperintensity.

### SES and LA Latent Variables

SES and LA showed a moderate positive correlation (Fig 1B). Spearman correlations among indicators were low to moderate except for the association between years of education and educational complexity, which showed a strong correlation (Fig 1C). Correlations between the extracted SES and LA scores with the indicators are in line with the factor loadings of the SES and LA constructs. They indicate that years of education is an extremely strong indicator of the SES latent variable, and the early life activity score is an extremely strong indicator of the LA latent variable.

In a linear regression model, we found that male sex showed a strong association with SES (Table 2). Additionally, APOE4 carriers had higher SES compared to non‐carriers. Aβ burden, WMH burden, age, and diagnosis (CU vs MCI) were not associated with SES. No significant association with LA was found.

**TABLE 2:** Results of the Linear Regression Analysis Examining Demographic Factors, Aβ Burden, and WMH Volume as Predictors of SES or LA

Predictor	SES		LA	
	Estimates (SE)	*p*	Estimates (SE)	*p*
Aβ	−0.04 (0.07)	0.601	0.02 (0.05)	0.648
WMH	−0.08 (0.07)	0.194	−0.05 (0.05)	0.331
Age	−0.12 (0.07)	0.082	−0.00 (0.05)	0.935
Sex (M)	0.79 (0.10)	<0.001	0.07 (0.09)	0.403
APOE4	0.33 (0.14)	0.019	0.15 (0.10)	0.157
Diagnosis (CU)	−0.23 (0.15)	0.11	−0.09 (0.11)	0.394

All variables included in the model were standardized.

Aβ = amyloid‐beta; APOE4 = apolipoprotein E4; CU = cognitively unimpaired; LA = lifestyle activities; M = male; SE = standard error; SES = socioeconomic status; WMH = white matter hyperintensity.

### Interactive Effects of SES and LA on the Relationship between Aβ, WMH, and Cognitive Decline

**FIGURE 2:**
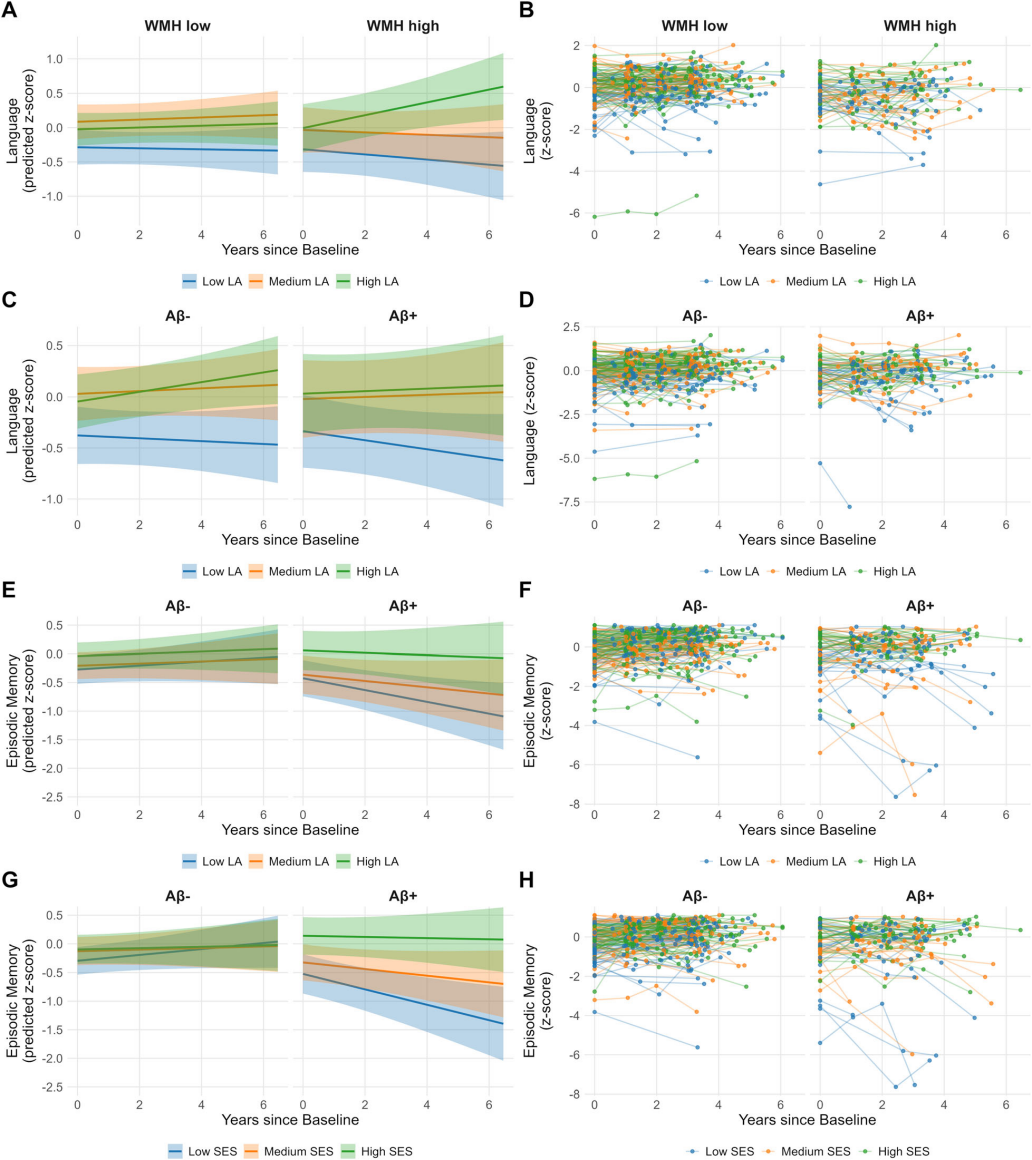
Lifestyle activities (LA) and socioeconomic status (SES) modify the effect of amyloid‐beta (Aβ)‐positron emission tomography (PET) and white matter hyperintensity (WMH) load on cognitive decline. The plot shows interactive effects of brain pathologies and SES or LA on longitudinal domain‐specific cognitive decline for which the *Score*_LA/SES_ × Aβ × *time* or *Score*_LA/SES_ × WMH × *time* interaction was significant. For the visualization, we used SES and LA tertiles and stratified the sample into low WMH (WMH < 66.6th percentile) and high WMH (WMH ≥ 66.6th percentile) or Aβ− (Centiloid <12) and Aβ+ (Centiloid ≥12) groups. Plots on the left show the predicted values for each group estimated from linear mixed‐effects models including random intercepts and slopes for each participant. Plots on the right show the raw z‐score changes over time for each participant. [Color figure can be viewed at www.annalsofneurology.org]

Linear mixed‐effects models showed that higher SES and LA were consistently associated with better baseline cognitive performance across all 4 cognitive domains (see Table 3 for detailed model estimates of terms of interest). In model A, which examined the longitudinal effects of SES, LA, Aβ, and WMH, higher baseline Aβ burden was associated with faster decline in episodic memory, language, and executive function scores, whereas a higher baseline WMH volume predicted faster decline in processing speed/attention. Neither SES nor LA were significant predictors of cognitive change over time in any domain. In model B, which examined the interaction *Score*_LA/SES_ × Aβ × time, we found significant interactions on language and episodic memory performance such that higher LA was associated with reduced cognitive decline in individuals with higher Aβ burden. Additionally, Aβ interacted with SES, such that lower SES was associated with a faster decline in episodic memory in the presence of elevated Aβ levels. A comparison between model B (3‐way interaction: *Score*_LA/SES_ × Aβ × *time*) and a simpler 2‐way interaction model (*Score*_LA_ × *time *+ Aβ × *time*) demonstrated that the more complex 3‐way interaction model provided a significantly better fit to the data (log‐likelihood ratio; language: χ^2^(2) = 21.7, *p* < 0.0001; episodic memory: χ^2^(2) = 7.4, *p* = 0.025). The same was true for the interaction with SES (episodic memory: χ^2^(2) = 20.7, *p* < 0.0001). Model C, which examined the interaction *Score*_LA/SES_ × WMH × *time*, indicated significant interactions between LA and WMH on language decline, such that higher LA was associated with reduced decline in individuals with higher WMH volume. The more complex 3‐way interaction model provided a significantly better fit to the data compared to the simpler 2‐way interaction model (χ^2^(2) = 6.67, *p* = 0.036). Interactions were still significant after FDR‐correction. Figure 2 illustrates the modifying effect of *Score*_LA/SES_ on cognitive outcomes for which significant interactions were found. Alcohol consumption, smoking status, living situation, comorbidity burden, and FHS‐CVD risk score were not associated with decline in any cognitive domain (Fig S1). Including these variables in the linear mixed effect models did not change the reported results. Subgroup analyses by APOE4 status and sex are reported in Table S2.

**TABLE 3:** Summary of Linear Mixed‐Effect Models Examining LA, SES, Aβ, and WMH Volume as Predictors of Longitudinal Cognitive Decline

Cognitive domain	Model	SES		LA	
		Estimates (SE)	*p*	Estimates (SE)	*p*
Language	Model A				
	*Score*_LA/SES_	0.136 (0.065)	0.037	0.137 (0.059)	0.021
	*Score*_LA/SES_ × *time*	0.010 (0.014)	0.484	0.012 (0.013)	0.363
	Aβ × *time*	−0.039 (0.016)	0.015	−0.039 (0.016)	0.014
	WMH × *time*	−0.026 (0.016)	0.103	−0.025 (0.016)	0.107
	Model B				
	*Score*_LA/SES_ × Aβ × *time*	0.008 (0.015)	0.602	0.044 (0.015)	0.004
	Model C				
	*Score*_LA/SES_ × WMH × *time*	0.012 (0.016)	0.445	0.042 (0.018)	0.018
Processing speed/attention	Model A				
	*Score*_LA/SES_	0.101 (0.047)	0.033	0.122 (0.042)	0.004
	*Score*_LA/SES_ × *time*	−0.006 (0.010)	0.532	0.002 (0.009)	0.844
	Aβ × *time*	−0.015 (0.011)	0.182	−0.015 (0.012)	0.199
	WMH × *time*	−0.030 (0.011)	0.008	−0.029 (0.011)	0.011
	Model B				
	*Score*_LA/SES_ × Aβ × *time*	0.005 (0.011)	0.644	0.007 (0.012)	0.537
	Model C				
	*Score*_LA/SES_ × WMH × *time*	0.007 (0.011)	0.542	0.009 (0.013)	0.481
Executive functions	Model A				
	*Score*_LA/SES_	0.200 (0.054)	<0.001	0.175 (0.049)	<0.001
	*Score*_LA/SES_ × *time*	−0.007 (0.014)	0.600	−0.002 (0.013)	0.858
	Aβ × *time*	−0.033 (0.016)	0.036	−0.033 (0.016)	0.040
	WMH × *time*	−0.020 (0.016)	0.194	−0.019 (0.016)	0.216
	Model B				
	*Score*_LA/SES_ × Aβ × *time*	0.002 (0.015)	0.875	0.026 (0.015)	0.094
	Model C				
	*Score*_LA/SES_ × WMH × *time*	0.021 (0.016)	0.197	0.030 (0.018)	0.098
Episodic memory	Model A				
	*Score*_LA/SES_	0.189 (0.062)	0.003	0.125 (0.057)	0.029
	*Score*_LA/SES_ × *time*	0.002 (0.015)	0.913	0.009 (0.014)	0.494
	Aβ × *time*	−0.101 (0.017)	<0.001	−0.101 (0.017)	<0.001
	WMH × *time*	0.029 (0.017)	0.090	0.03 (0.017)	0.076
	Model B				
	*Score*_LA/SES_ × Aβ × *time*	0.053 (0.016)	0.001	0.044 (0.016)	0.006
	Model C				
	*Score*_LA/SES_ × WMH × *time*	0.013 (0.019)	0.474	0.006 (0.021)	0.758

Aβ = amyloid‐beta; LA = lifestyle activities; SE = standard error; SES = socioeconomic status; WMH = white matter hyperintensity.

As both Aβ and WMH interacted with LA to predict longitudinal language performance, we tested whether they contribute independently to the prediction by including the 2 interaction terms in the same model. The results indicate that both interactions contribute independent information to longitudinal language performance (WMH × *Score*_LA_ × *time*: β = 0.042 (0.017), *p* = 0.013, Aβ × *Score*_LA_ × *time *= 0.046 (0.016), *p* = 0.014).

When we defined the LA construct using only early‐ and mid‐life activity indicators, the results were consistent with those reported above (Table S3). In contrast, when LA was defined solely by current CA and PA indicators, the 3‐way interactions between LA, Aβ/WMH burden, and time were no longer significantly associated with declines in language or episodic memory (Table S4). However, individuals with higher current activity scores tended to perform better across all cognitive domains at baseline. These findings suggest that although higher LA activity levels in early‐, mid‐, and late‐life are associated with superior baseline cognition, the moderating effects on cognitive decline are primarily driven by early‐ and mid‐life activities. Finally, removing APOE4 status as a covariate (Table S5) or excluding years of education from the SES construct (Table S6) did not substantially alter the reported results.

### LA as Potential Mediator between SES and Cognitive Decline

**FIGURE 3:**
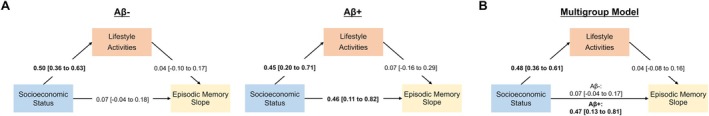
Lifestyle activities do not mediated the association between socioeconomic status and episodic memory decline. A, shows separate path estimates for Aβ− and Aβ+ groups. B, shows the final multigroup model, in which only paths that differ significantly between the groups are estimated separately. [Color figure can be viewed at www.annalsofneurology.org]

Because both SES and LA interacted with Aβ burden to predict episodic memory decline, we used a moderated mediation model to investigate whether LA statistically mediates the effect of SES on episodic memory decline. The model reveals strong associations between *Score*_SES_ and *Score*_LA_ in both Aβ+ and Aβ− groups, but *Score*_LA_ does not mediate the relationship between *Score*_SES_ and episodic memory change (Fig 3A). Instead, *Score*_SES_ emerges as the primary factor that influences cognitive decline in Aβ+ individuals, whereas the effect of Score_LA_ becomes non‐significant once *Score*_SES_ is taken into account. The final multigroup model (Fig 3B) confirmed that the path from SES to episodic memory slope differed significantly between the 2 groups (*p* = 0.010), whereas constraining either of the other 2 paths to be equal across groups did not result in a significant decrease in model fit (*p* > 0.74), and therefore, these paths were estimated as a single parameter.

### Gray Matter Atrophy as Mediator between Aβ and Cognitive Decline

**FIGURE 4:**
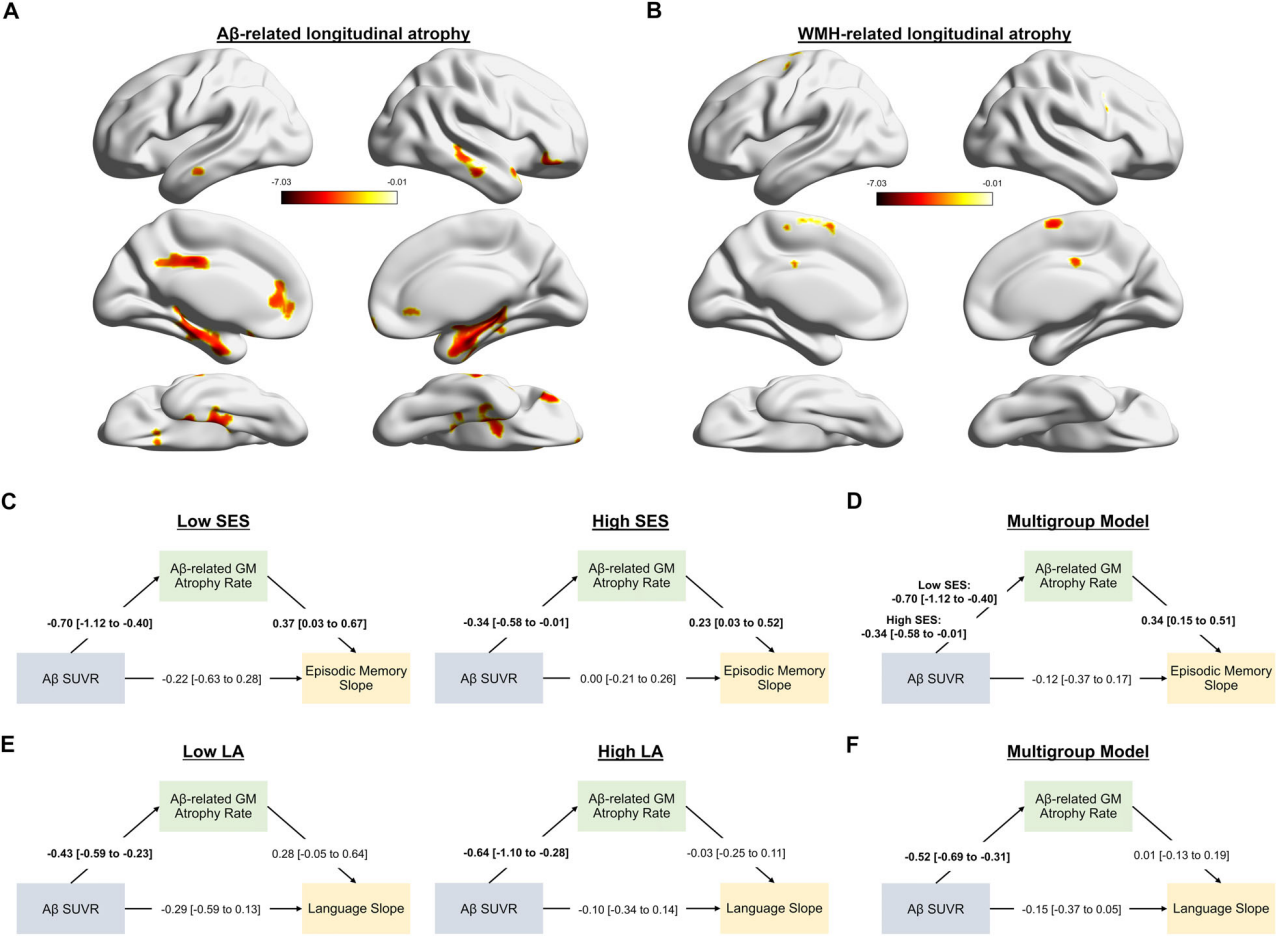
The mediating effect of gray matter (GM) atrophy is more pronounced in individuals with high socioeconomic status (SES) because of faster amyloid‐beta (Aβ)‐related GM atrophy rates. A and B display Aβ standardized uptake value ratio (SUVR) and white matter hyperintensity (WMH)‐related atrophy clusters that survived random field theory correction (voxel‐level *p* < 0.001; cluster‐level *p* < 0.05). C, shows separate path estimates for individuals with low and high SES, with episodic memory slope as the outcome. D, shows the final multigroup model, in which only paths that differ significantly between groups are estimated separately. Similarly, E, shows path estimates for language slope as the outcome across SES groups. The final model in F indicates no significant differences in path estimates between the groups. [Color figure can be viewed at www.annalsofneurology.org]

Figure 4A shows the results of the longitudinal voxel‐wise analysis of gray matter atrophy. Higher Aβ burden was associated with faster rates of gray matter atrophy in medial temporal, parietal, and frontal regions. In contrast, WMH volume predicted atrophy rates only in a few clusters. Given their location and limited extent, these WMH‐related atrophy clusters were not examined further. Neither SES nor LA were associated with the rate of gray matter atrophy. Unthresholded *t*‐value maps for Aβ‐, WMH‐, SES‐ and LA‐related atrophy are shown in Figure S2. At baseline, we found no associations between either SES or LA and gray matter volume, Aβ burden was associated with reduced gray matter volume only in the left medial temporal lobe, whereas higher WMH volume was associated with lower gray matter volume in multiple regions distributed across the brain (Fig S3).

Based on our previous findings that Aβ interacts with SES to predict episodic memory decline and with LA to predict language decline, we next investigated whether the Aβ‐related gray matter atrophy rate mediates these interactions. As shown in Figure 4C, higher Aβ SUVR predicted faster rates of gray matter atrophy in both the low and the high SES groups, but the association was notably stronger in the low SES group. In both groups, the rate of Aβ‐related gray matter atrophy, but not the direct path from Aβ SUVR, was associated with episodic memory slope. When formally testing for group differences in the path estimates, we found a significant difference in the path from Aβ SUVR to gray matter atrophy rate, but not in the other 2 paths. The final multigroup model is presented in Figure 4D. These findings were largely consistent in subsamples of men and women, although the association between gray matter atrophy rate and episodic memory slope reached significance only among men (Fig S4). In contrast, neither Aβ SUVR nor the Aβ‐related gray matter atrophy rate were associated with language decline in either the low or high LA group, and none of the paths differed significantly between these groups (Fig 4E,F).

## Discussion

In this longitudinal study, we used self‐reported data of SES and LA across early life, mid‐life, and late life, to examine the effects of SES and LA on regional gray matter atrophy rates and domain‐specific cognitive decline. We first observed that individuals with higher SES and LA scores showed signs of cognitive reserve, as they performed better cognitively at baseline and experienced less cognitive decline for a given level of Aβ pathology and white matter lesion volume. Additionally, we found evidence of greater brain maintenance in individuals with higher SES, as they showed reduced rates of Aβ‐related gray matter atrophy. These findings support several important conclusions. First, a lifestyle high in cognitive, social, and physical activities as well as a higher SES may delay the onset of cognitive symptoms in individuals at increased risk of developing dementia. Second, interventions targeting SES‐related risk factors to mitigating Aβ‐related decline may need to begin early, before neurodegenerative occur. This is supported by our observation that lower SES amplified the impact of Aβ on gray matter atrophy rate, but it did not affect the relationship between atrophy and memory decline. Third, multiple and distinct resilience mechanisms may be involved, as the effects of LA and WMH burden on cognitive decline appear to operate independently of gray matter atrophy. A better understanding of how these resilience factors offer protection can help reduce SES‐related inequalities in dementia risk and support the development of strategies that use modifiable factors to prevent cognitive decline.

A major novelty of our study lies in the comprehensive integration of multiple data domains including longitudinal measurement of gray matter atrophy and domain‐specific cognitive decline, which allowed us to more precisely determine how and when LA and SES exert their protective effects. Although both factors were associated with better baseline cognitive performance across all domains, they showed distinct longitudinal effects. LA was associated with a reduced decline in language, regardless of whether its interaction with Aβ or WMH was examined, suggesting that mentally stimulating activities may strengthen language neural networks^29^ by reinforcing vocabulary and syntactic knowledge regardless of the underlying brain pathology. A longer follow up period may be required to observe the same interactions for executive functions, for which a comparable, non‐significant trend was observed. In contrast, higher SES was specifically associated with reduced Aβ‐related memory decline, an effect that was mediated by gray matter atrophy. The lack of association between WMH burden and gray matter atrophy suggests that SES and LA may confer resilience through distinct, pathology‐specific pathways. For WMH burden, disruption of white matter tracts may represent a more relevant mediating mechanism.^30^, ^31^

Importantly, SES did not modify the association between gray matter atrophy and episodic memory decline, but exerted its influence earlier in the hypothetical disease process by attenuating the rate of Aβ‐related gray matter atrophy. The finding that gray matter atrophy was associated with episodic memory decline regardless of SES may suggest that interventions aimed at enhancing resilience may need to occur early, before neurodegenerative processes have significantly advanced. Aβ‐PET burden is known to contribute to gray matter atrophy primarily through its association with tau‐PET burden,^32^, ^33^ hence, a plausible explanation for our findings is that SES modulates the relationship between Aβ and tau pathology. Such modulation effects have been reported for several factors, including vascular health,^34^ functional brain connectivity,^35^, ^36^ sex‐related factors,^37^ and microglial activation.^38^ Whether SES operates as an independent resilience factor or reflects one or more of these underlying mechanisms remains an open question for future research. Our sensitivity analyses suggest that sex differences are unlikely to account for the observed effects, as similar associations were found in both men and women. Alternatively, prior work has shown that education can moderate the relationship between tau‐PET burden and cortical atrophy.^12^ However, in that study, higher levels of education did not show a protective effect longitudinally, instead, individuals with more years of education showed faster rates of gray matter atrophy at a given level of tau burden. The inclusion of only Aβ‐positive individuals with MCI and AD, as opposed to our cohort, which is a community‐based sample of comparatively healthy participants, is a key difference between that study and ours that may explain the divergent findings. Together, these results suggest that individuals with high SES experience slower Aβ‐related atrophy during early pathological or preclinical stages, but exhibit a faster atrophy after clinical presentations of AD. This pattern has previously been observed for cognitive performance in the context of cognitive reserve,^39^, ^40^ and it appears to also apply for brain atrophy in the context of brain maintenance.

A certain level of brain pathology likely needs to be present to observe meaningful cognitive decline—and, consequently, the modifying effects of LA or SES—within a relatively short follow‐up period such as the 3 to 4 years in our study. Although higher LA and SES were associated with better baseline cognitive performance, no effects on cognition over time were observed when we modeled only their direct interactions with time (model A). However, when we included their 3‐way interactions with brain pathology (models B and C), longitudinal effects of LA and SES on cognitive changes became evident, indicating that, depending on the cohort, modifiable effects on cognitive trajectories may not be recognizable without considering these interactions.

Unhealthy lifestyles are frequently proposed as mediators between SES and health related outcomes, but the contribution varies according to geographical region, population characteristics, and study design.^27^, ^28^ Two large prospective cohort studies investigated the mediating effect of LA between SES and dementia risk found that lifestyle mediates only a small proportion of the socioeconomic inequity in dementia risk in United States and United Kingdom older adults.^11^, ^41^ Similarly, in a large cohort of Black and White older adults, up to 25% of the association between education and dementia was mediated through mid‐life vascular risk factors, including systolic blood pressure, fasting glucose, body mass index, and smoking.^42^ Our findings align with these observations to some degree, as not all modifying effects were attributable solely to LA. It is possible that factors associated with SES, but unrelated to lifestyle, such as a higher prevalence of traumatic brain injury or greater exposure to air pollution among individuals with low SES, may exacerbate the observed effects.^5^ Another possibility may be that SES‐related brain changes are established as early as childhood, which may allow a better copying with Aβ‐related brain changes.^43^

Our SES and LA scores were derived primarily from early life and mid‐life measures, which is particularly relevant given substantial evidence that many risk factors are most detrimental when present in mid‐life.^5^ Importantly, although both early‐/mid‐life LA and current LA scores were associated with baseline cognitive performance when considered separately, the influence on cognitive decline over time was primarily related to early‐/mid‐life activities, but not to current activities. Lifestyle interventions may, therefore, be more effective when introduced earlier in life. Nevertheless, intervention studies have shown that multidomain lifestyle interventions can improve cognitive performance also in adults over 60,^44^ regardless of a wide range of baseline characteristics.^45^ The effectiveness of such interventions may, however, be limited if contextual factors are not taken into account. Barriers such as lack of time, access issues, financial costs, and restrictions in the physical environment have been found to be among the key obstacles to adopting healthy behaviors in mid‐life.^46^ Risk reduction strategies need to consider these key issues. A more comprehensive approach, which considers community resources, social support, healthcare access, and environmental conditions, is likely to be more effective in reducing disparities in dementia risk and promoting healthier behaviors across different socioeconomic groups.^10^

Several limitations of our study warrant consideration. Although this is a prospective study, reversed causation may still be a concern. Physical and cognitive activity may decline years before a dementia diagnosis.^47^, ^48^ However, it is worth noting that many of our measures were drawn from early life and mid‐life, and current activities alone did not show the same modifying effect. Furthermore, our sample consists of primarily White, highly educated individuals, which limits the generalizability to other ethnic groups and populations with different levels of health awareness or access. Information on SES and LA was self‐reported, although measurement uncertainties may be mitigated by our latent variable approach. The method also has the advantage that it accounts for the health behaviors and socioeconomic determinants that tend to cluster.^49^ However, it does not fully disentangle the unique contributions of individual exposures (ie, physical vs cognitive activities).

In conclusion, higher LA reduced the detrimental effects of Aβ pathology and white matter lesions on language decline likely independent of gray matter atrophy. In contrast, higher SES reduced the detrimental effects of Aβ pathology on episodic memory decline by reducing Aβ‐related gray matter atrophy. Our results support the idea that sociobehavioral factors enhance cognitive resilience against brain pathologies.^50^ More research is needed to determine the optimal timing and duration of interventions and to better understand the underlying factors contributing to socioeconomic disparities in dementia.

## Author Contributions

D.B., R.M.N., C.H., A.G., and V.T. contributed to the conception and design of the study; D.B., M.W., A.B., A.S., S.S., K.T., B.v.R., E.G., A.G., and V.T. acquisition and analysis of data. D.B., A.G., and V.T. contributed to drafting the text and preparing the figures.

## Potential Conflicts of Interest

Nothing to report.

## Supporting information


**Data S1.** Supporting Information.

## Data Availability

The data that support the findings of this study are available from the corresponding author on reasonable request.
