# Supplementary material for: Protective Effects of Socioeconomic Status and Lifestyle on Amyloid‐ and White Matter Hyperintensity‐Related Longitudinal Brain Atrophy and Cognitive Decline
Source: Ann Neurol. 2025 Aug 9;98(6):1222–36. doi: 10.1002/ana.70022 (PMC12682946; doi:10.1002/ana.70022)
Supplement: Supplementary file 1 — Data S1. Supporting Information. [file ANA-98-1222-s001.docx]

**Supplementary Materials**

**Figure S1. Potential confounders were not associated with cognitive decline in any cognitive domain.**

The estimates in the plot represent the beta coefficients with 95% confidence intervals for the *confounder* × time interaction from separate linear mixed-effects models, adjusted for age, sex, APOE4 carrier status, and their interactions with time.


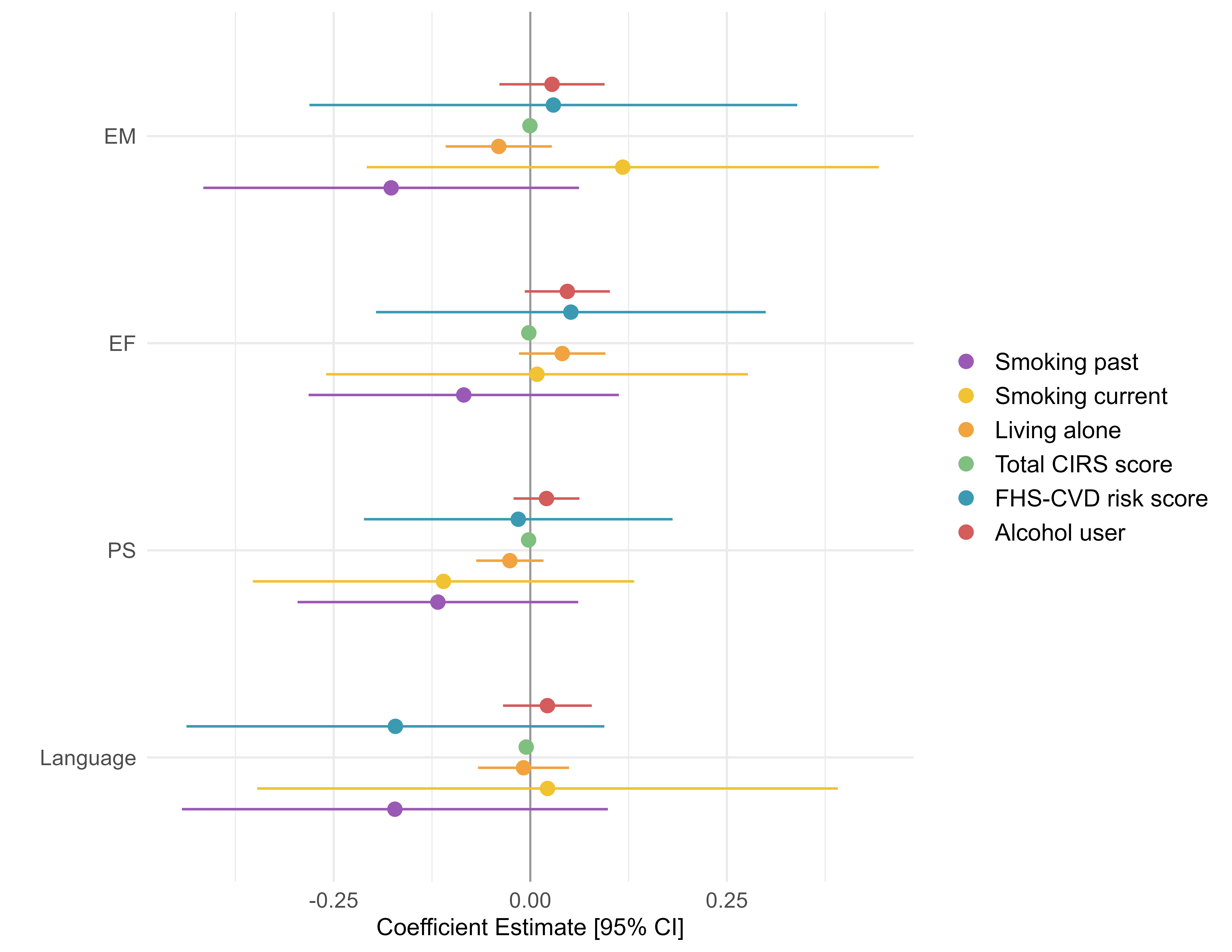


**Figure S2. Longitudinal gray matter atrophy.**

The plots display unthresholed voxelwise t-statistic maps derived from linear regression analyses, adjusted for age, sex, and total intracranial volume (TIV). Outlined in black are clusters that survived random field theory correction with p <0.001 at voxel level and cluster threshold set at p <0.05.


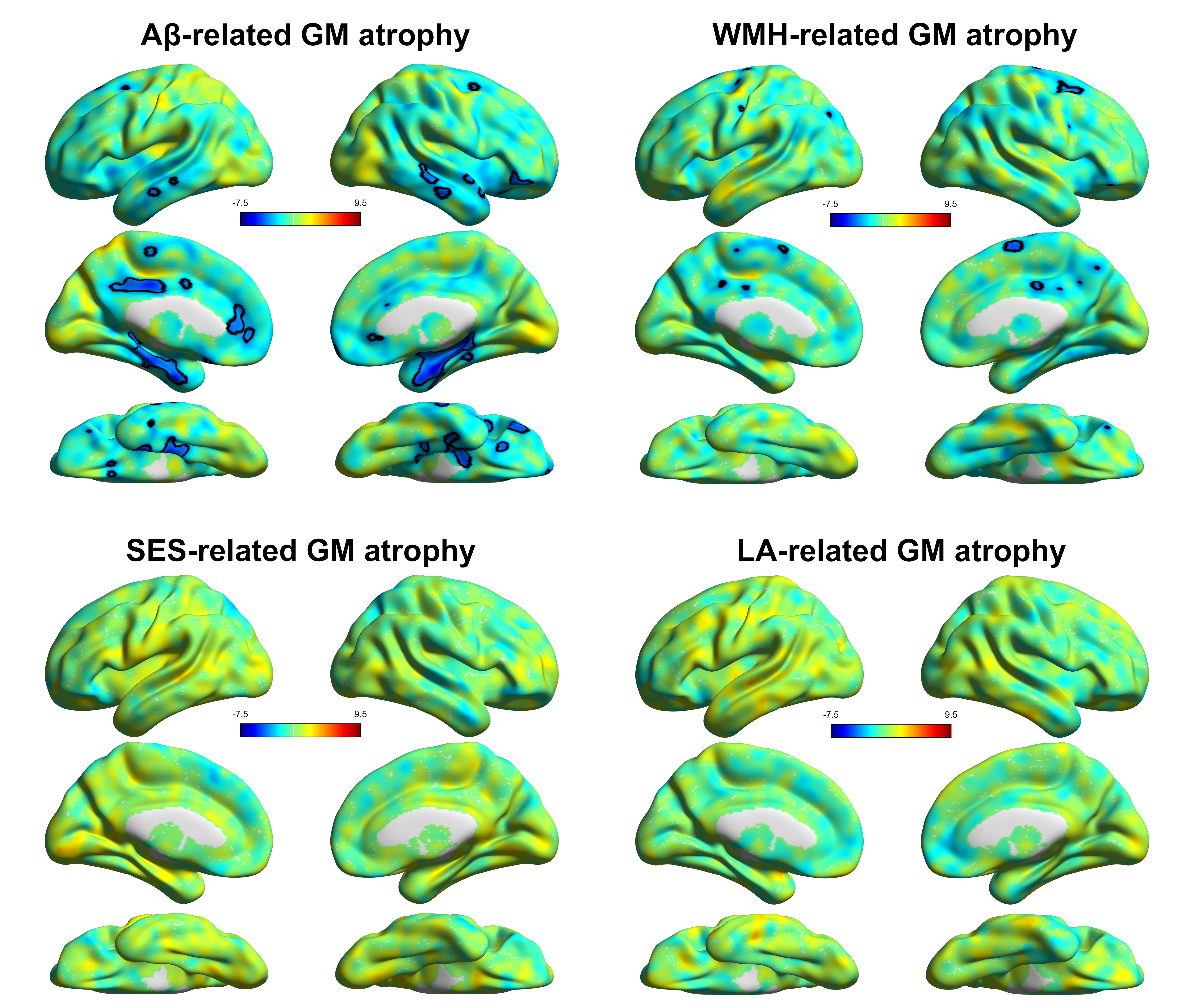


**Figure S3. Crosssectional voxelwise associations between gray matter (GM) volume and variables of interest.**

The plots display clusters that survived random field theory correction. Models were adjusted for age, sex, and total intracranial volume (TIV).


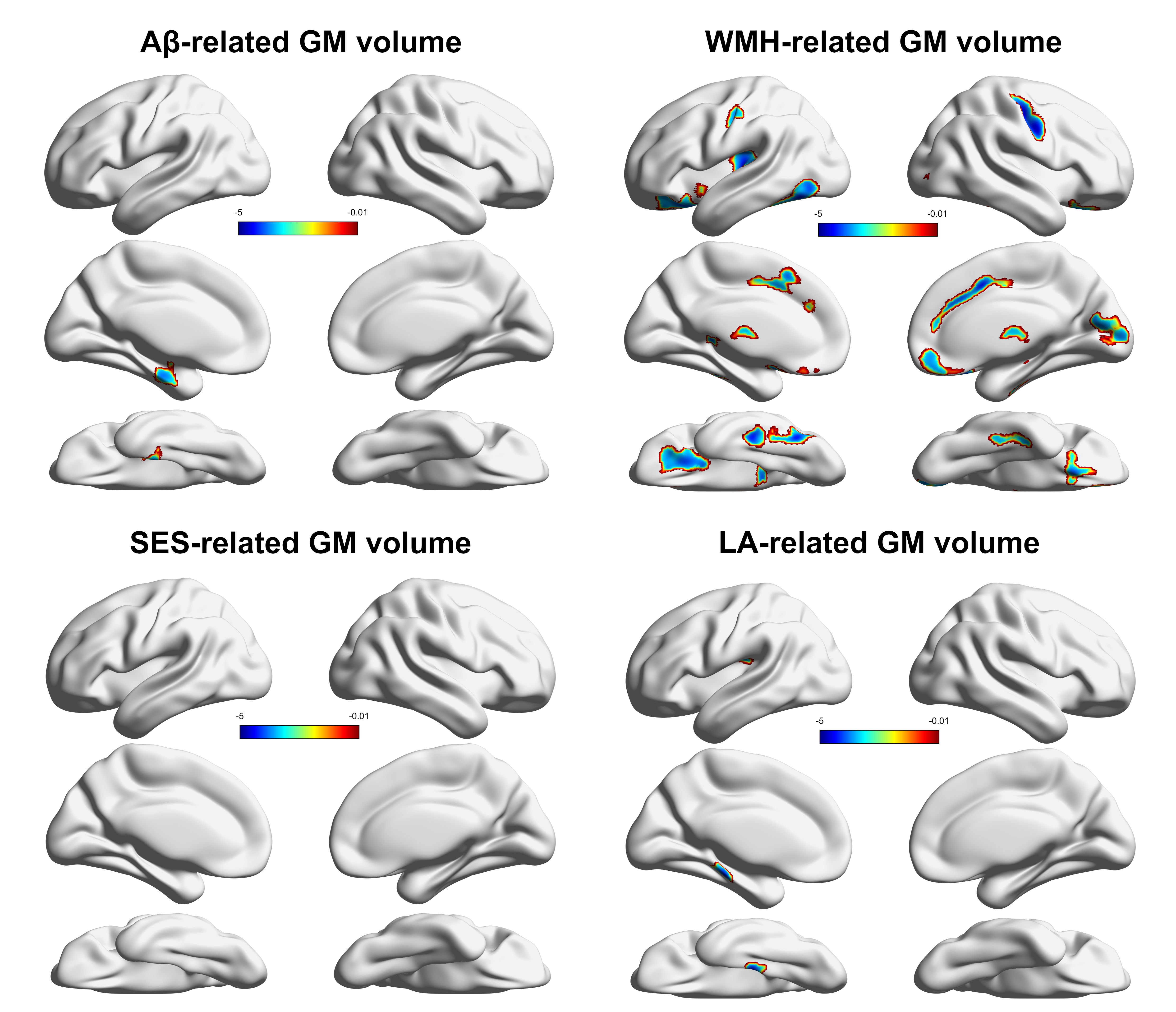


**Figure S4. Moderated mediation model for subgroups of men and women.**

Subgroups of men and women were created using a median split within each sample. The moderated mediation models were adjusted for age and APOE4 status. Estimates were based on 1000 bootstrap resamples.





**Table S1.** Cohort characteristics of participants with longitudinal T1w MRI.

|  | Participants (N = 181) |
| --- | --- |
| Age at Baseline, mean years (SD) [range] | 67 (8.2)  [51-90] |
| Female sex, N (%) | 85 (47.0) |
| MMSE, mean (SD) | 29.2 (1.1) |
| MCI, N (%) | 42 (23.2) |
| APOE4 carriers, N (%) | 40 (22.1) |
| Aβ-PET burden, mean SUVR (SD) | 1.27 (0.22) |
| Centiloid >12, N (%) | 60 (33.1) |
| WMH volume, median % of ICV (IQR), (NA = 9) | 0.17 [0.08-0.35] |
| Number of NP assessments, 2/3/4/5, N | 32/41/81/27 |
| NP Follow-up years, median years (IQR) | 3.5 (3.2 to 4.5) |
| T1 MRI Follow-up years, median years (IQR) | 3.4 (3.1 to 4.3) |
| Baseline total CIRS score, median (IQR) | 6 [4-9] |
| Baseline FHS-CVD risk score (in %), mean (SD) | 18 (0.12) |
| Alcohol use, N (%) | 34 (19.9) |
| Smoker (past or present), N (%) | 91 (50.3) |
| **SES indicators** |  |
| Years of education, mean (SD) | 15.4 (2.8) |
| Net income, mean (SD), (NA = 22) | 4.7 (1.3) |
| Education score, mean (SD), (NA = 1) | 12.0 (5.8) |
| Occupation score, mean (SD), (NA = 1) | 11.3 (2.6) |
| **LA indicators** |  |
| Current CA, mean (SD), (NA = 1) | 23.0 (7.2) |
| Current PA, mean (SD), (NA = 1) | 13.5 (5.8) |
| Early life activities score, mean (SD) | 23.3 (4.4) |
| Mid-life activities score, mean (SD) | 23.5 (4.5) |

Abbreviations: APOE4 = apolipoprotein E4, CA = cognitive activity, CIRS = cumulative illness rating scale, FHS-CVD = Framingham Heart Study cardiovascular disease, IQR = interquartile range, MCI = mild cognitive impairment, SD = standard deviation, MMSE = mini-mental state examination, NA = not available, NP = neuropsychological, PA = physical activity, PET = positron emission tomography, SUVR = standardized uptake value ratio

**Table S2. Summary of linear mixed-effect models examining LA, SES, Aβ, and WMH volume as predictors of longitudinal cognitive decline in sex and APOE4 status stratified samples.**

|  | **Term** | **SES** | | | **LA** | | |
| --- | --- | --- | --- | --- | --- | --- | --- |
|  |  | **estimate** | **std.error** | **p value** | **estimate** | **std.error** | **p value** |
| Male (N = 117) | |  |  |  |  |  |  |
| Language | Score_LA/SES_*WMH*time | -0.032 | 0.022 | 0.15 | 0.01 | 0.024 | 0.693 |
|  | Score_LA/SES_ *Aβ*time | -0.049 | 0.023 | **0.031** | 0.012 | 0.023 | 0.604 |
| PS/Attention | Score_LA/SES_*WMH*time | -0.024 | 0.017 | 0.159 | 0.013 | 0.019 | 0.493 |
|  | Score_LA/SES_ *Aβ*time | -0.002 | 0.017 | 0.886 | 0.017 | 0.018 | 0.368 |
| Executive Function | Score_LA/SES_*WMH*time | 0.011 | 0.025 | 0.656 | 0.026 | 0.027 | 0.344 |
|  | Score_LA/SES_ *Aβ*time | -0.021 | 0.024 | 0.386 | 0.048 | 0.023 | **0.039** |
| Episodic Memory | Score_LA/SES_*WMH*time | -0.009 | 0.026 | 0.737 | 0.039 | 0.025 | 0.127 |
|  | Score_LA/SES_ *Aβ*time | 0.018 | 0.025 | 0.486 | 0.059 | 0.025 | **0.022** |
| Female (N = 104) | |  |  |  |  |  |  |
| Language | Score_LA/SES_*WMH*time | 0.063 | 0.033 | 0.057 | 0.067 | 0.026 | **0.012** |
|  | Score_LA/SES_ *Aβ*time | 0.044 | 0.03 | 0.144 | 0.075 | 0.022 | **0.001** |
| PS/Attention | Score_LA/SES_*WMH*time | 0.014 | 0.022 | 0.521 | 0.001 | 0.018 | 0.937 |
|  | Score_LA/SES_ *Aβ*time | -0.006 | 0.021 | 0.767 | -0.003 | 0.016 | 0.84 |
| Executive Function | Score_LA/SES_*WMH*time | 0.057 | 0.029 | 0.051 | 0.028 | 0.024 | 0.236 |
|  | Score_LA/SES_ *Aβ*time | 0.029 | 0.027 | 0.282 | 0.019 | 0.02 | 0.356 |
| Episodic Memory | Score_LA/SES_*WMH*time | 0.021 | 0.039 | 0.593 | 0.015 | 0.031 | 0.626 |
|  | Score_LA/SES_ *Aβ*time | 0.064 | 0.029 | **0.029** | 0.01 | 0.022 | 0.662 |
| APOE4 carrier (N = 52) | |  |  |  |  |  |  |
| Language | Score_LA/SES_*WMH*time | 0.065 | 0.04 | 0.105 | 0.204 | 0.063 | **0.001** |
|  | Score_LA/SES_ *Aβ*time | 0.051 | 0.029 | 0.077 | 0.115 | 0.025 | **<0.001** |
| PS/Attention | Score_LA/SES_*WMH*time | 0.027 | 0.024 | 0.257 | 0.074 | 0.042 | 0.083 |
|  | Score_LA/SES_ *Aβ*time | 0.017 | 0.019 | 0.382 | 0.016 | 0.02 | 0.407 |
| Executive Function | Score_LA/SES_*WMH*time | 0.018 | 0.038 | 0.645 | 0.083 | 0.063 | 0.186 |
|  | Score_LA/SES_ *Aβ*time | 0.026 | 0.03 | 0.384 | 0.033 | 0.028 | 0.244 |
| Episodic Memory | Score_LA/SES_*WMH*time | 0.06 | 0.036 | 0.097 | 0.181 | 0.056 | **0.002** |
|  | Score_LA/SES_ *Aβ*time | 0.048 | 0.027 | 0.078 | 0.043 | 0.028 | 0.123 |
| APOE4 non-carrier (N = 169) | |  |  |  |  |  |  |
| Language | Score_LA/SES_*WMH*time | 0.005 | 0.018 | 0.793 | 0.03 | 0.018 | 0.105 |
|  | Score_LA/SES_ *Aβ*time | -0.02 | 0.02 | 0.321 | 0.01 | 0.02 | 0.622 |
| PS/Attention | Score_LA/SES_*WMH*time | 0.001 | 0.013 | 0.958 | 0.001 | 0.014 | 0.954 |
|  | Score_LA/SES_ *Aβ*time | 0.004 | 0.015 | 0.788 | 0.011 | 0.016 | 0.494 |
| Executive Function | Score_LA/SES_*WMH*time | 0.022 | 0.018 | 0.23 | 0.024 | 0.019 | 0.193 |
|  | Score_LA/SES_ *Aβ*time | -0.005 | 0.019 | 0.792 | 0.037 | 0.02 | 0.063 |
| Episodic Memory | Score_LA/SES_*WMH*time | 0.001 | 0.021 | 0.968 | -0.018 | 0.022 | 0.418 |
|  | Score_LA/SES_ *Aβ*time | 0.058 | 0.021 | **0.007** | 0.054 | 0.022 | **0.014** |

**Table S3. Summary of linear mixed-effect models examining the interaction between LA without current activities, Aβ, and WMH volume as predictors of longitudinal cognitive decline.**

Scorepast_LA indicates that LA was derived from a latent variable that only included the Early life Activities and Mid-life Activities indicators.

| Cognitive Domain | Model | Past LA | | |
| --- | --- | --- | --- | --- |
|  |  | Estimates | SE | p value |
| Language | **Model A:** |  |  |  |
|  | Score_past_LA_ | 0.125 | 0.059 | **0.036** |
|  | Score_past_LA_*time | 0.009 | 0.013 | 0.467 |
|  | WMH*time | -0.026 | 0.016 | 0.104 |
|  | Aβ*time | -0.039 | 0.016 | **0.014** |
|  | **Model B:** |  |  |  |
|  | Score_past_LA_*Aβ*time | 0.043 | 0.015 | **0.005** |
|  | **Model C:** |  |  |  |
|  | Score_past_LA_*WMH*time | 0.039 | 0.017 | **0.025** |
| Processing Speed/Attention | **Model A:** |  |  |  |
|  | Score_past_LA_ | 0.117 | 0.042 | **0.006** |
|  | Score_past_LA_*time | 0.003 | 0.009 | 0.708 |
|  | WMH*time | -0.028 | 0.011 | **0.012** |
|  | Aβ*time | -0.015 | 0.012 | 0.2 |
|  | **Model B:** |  |  |  |
|  | Score_past_LA_*Aβ*time | 0.004 | 0.012 | 0.714 |
|  | **Model C:** |  |  |  |
|  | Score_past_LA_*WMH*time | 0.012 | 0.013 | 0.346 |
| Executive Functions | **Model A:** |  |  |  |
|  | Score_past_LA_ | 0.173 | 0.049 | **0.001** |
|  | Score_past_LA_*time | -0.005 | 0.013 | 0.724 |
|  | WMH*time | -0.019 | 0.016 | 0.209 |
|  | Aβ*time | -0.033 | 0.016 | **0.039** |
|  | **Model B:** |  |  |  |
|  | Score_past_LA_*Aβ*time | 0.024 | 0.015 | 0.115 |
|  | **Model C:** |  |  |  |
|  | Score_past_LA_*WMH*time | 0.031 | 0.018 | 0.08 |
| Episodic Memory | **Model A:** |  |  |  |
|  | Score_past_LA_ | 0.1 | 0.057 | 0.082 |
|  | Score_past_LA_*time | 0.009 | 0.014 | 0.506 |
|  | WMH*time | 0.03 | 0.017 | 0.078 |
|  | Aβ*time | -0.1 | 0.017 | **<0.001** |
|  | **Model B:** |  |  |  |
|  | Score_past_LA_*Aβ*time | 0.036 | 0.016 | **0.024** |
|  | **Model C:** |  |  |  |
|  | Score_past_LA_*WMH*time | 0.002 | 0.02 | 0.921 |

**Table S4.** **Summary of linear mixed-effect models examining the interaction between LA without past activities, Aβ, and WMH volume as predictors of longitudinal cognitive decline.**

Scorecurrent_CA/PA indicates that LA was derived from a latent variable that only included the Current CA and Current PA indicators.

| Cognitive Domain | Model | Current LA | | |
| --- | --- | --- | --- | --- |
|  |  | Estimates | SE | p value |
| Language | **Model A:** |  |  |  |
|  | Score_current_CA/PA_ | 0.105 | 0.06 | 0.08 |
|  | Score_CA/PA_*time | 0.01 | 0.013 | 0.446 |
|  | WMH*time | -0.027 | 0.016 | 0.084 |
|  | Aβ*time | -0.04 | 0.016 | **0.013** |
|  | **Model B:** |  |  |  |
|  | Score_current_CA/PA_*Aβ*time | 0.004 | 0.014 | 0.796 |
|  | **Model C:** |  |  |  |
|  | Score_current_CA/PA_*WMH*time | -0.008 | 0.017 | 0.632 |
| Processing Speed/Attention | **Model A:** |  |  |  |
|  | Score_current_CA/PA_ | 0.085 | 0.043 | 0.051 |
|  | Score_CA/PA_*time | -0.004 | 0.009 | 0.628 |
|  | WMH*time | -0.029 | 0.011 | **0.01** |
|  | Aβ*time | -0.015 | 0.011 | 0.193 |
|  | **Model B:** |  |  |  |
|  | Score_current_CA/PA_*Aβ*time | 0.012 | 0.01 | 0.257 |
|  | **Model C:** |  |  |  |
|  | Score_current_CA/PA_*WMH*time | -0.035 | 0.012 | **0.004** |
| Executive Functions | **Model A:** |  |  |  |
|  | Score_current_CA/PA_ | 0.001 | 0.051 | 0.985 |
|  | Score_CA/PA_*time | 0.014 | 0.013 | 0.292 |
|  | WMH*time | -0.02 | 0.015 | 0.206 |
|  | Aβ*time | -0.033 | 0.016 | **0.037** |
|  | **Model B:** |  |  |  |
|  | Score_current_CA/PA_*Aβ*time | 0.024 | 0.014 | 0.088 |
|  | **Model C:** |  |  |  |
|  | Score_current_CA/PA_*WMH*time | -0.02 | 0.017 | 0.245 |
| Episodic Memory | **Model A:** |  |  |  |
|  | Score_current_CA/PA_ | 0.178 | 0.057 | **0.002** |
|  | Score_CA/PA_*time | -0.006 | 0.014 | 0.653 |
|  | WMH*time | 0.029 | 0.017 | 0.086 |
|  | Aβ*time | -0.101 | 0.017 | **<0.001** |
|  | **Model B:** |  |  |  |
|  | Score_current_CA/PA_*Aβ*time | 0.012 | 0.014 | 0.395 |
|  | **Model C:** |  |  |  |
|  | Score_current_CA/PA_*WMH*time | -0.007 | 0.019 | 0.725 |

**Table S5.** **Summary of linear mixed-effect models without adjustment for APOE4 status.**

| Cognitive Domain | Model | SES | | LA | |
| --- | --- | --- | --- | --- | --- |
|  |  | Estimates (SE) | p value | Estimates (SE) | p value |
| Language | **Model A:** |  |  |  |  |
|  | Score_LA/SES_ | 0.149 (0.064) | **0.021** | 0.145 (0.059) | **0.015** |
|  | Score_LA/SES_*time | 0.010 (0.013) | 0.463 | 0.012 (0.013) | 0.349 |
|  | Aβ*time | -0.038 (0.015) | **0.012** | -0.039 (0.015) | **0.012** |
|  | WMH*time | -0.026 (0.016) | 0.103 | -0.025 (0.015) | 0.108 |
|  | **Model B:** |  |  |  |  |
|  | Score_LA/SES_*Aβ*time | 0.008 (0.015) | 0.593 | 0.043 (0.015) | **0.005** |
|  | **Model C:** |  |  |  |  |
|  | Score_LA/SES_*WMH*time | 0.012 (0.016) | 0.438 | 0.043 (0.018) | **0.015** |
| Processing Speed/  Attention | **Model A:** |  |  |  |  |
|  | Score_LA/SES_ | 0.112 (0.046) | **0.017** | 0.130 (0.042) | **0.002** |
|  | Score_LA/SES_*time | -0.007 (0.010) | 0.460 | 0.001 (0.009) | 0.919 |
|  | Aβ*time | -0.017 (0.011) | 0.122 | -0.017 (0.011) | 0.125 |
|  | WMH*time | -0.030 (0.011) | **0.008** | -0.028 (0.011) | **0.012** |
|  | **Model B:** |  |  |  |  |
|  | Score_LA/SES_*Aβ*time | 0.005 (0.011) | 0.661 | 0.008 (0.012) | 0.513 |
|  | **Model C:** |  |  |  |  |
|  | Score_LA/SES_*WMH*time | 0.007 (0.011) | 0.547 | 0.011 (0.013) | 0.410 |
| Executive Functions | **Model A:** |  |  |  |  |
|  | Score_LA/SES_ | 0.198 (0.053) | **<0.001** | 0.175 (0.049) | **<0.001** |
|  | Score_LA/SES_*time | -0.005 (0.014) | 0.711 | -0.001 (0.013) | 0.942 |
|  | Aβ*time | -0.029 (0.015) | 0.056 | -0.029 (0.015) | 0.059 |
|  | WMH*time | -0.021 (0.015) | 0.186 | -0.020 (0.015) | 0.208 |
|  | **Model B:** |  |  |  |  |
|  | Score_LA/SES_*Aβ*time | 0.002 (0.016) | 0.892 | 0.023 (0.015) | 0.132 |
|  | **Model C:** |  |  |  |  |
|  | Score_LA/SES_*WMH*time | 0.021 (0.016) | 0.198 | 0.029 (0.018) | 0.106 |
| Episodic Memory | **Model A:** |  |  |  |  |
|  | Score_LA/SES_ | 0.191 (0.061) | **0.002** | 0.129 (0.057) | **0.024** |
|  | Score_LA/SES_*time | 0.003 (0.015) | 0.842 | 0.010 (0.014) | 0.459 |
|  | Aβ*time | -0.098 (0.016) | **<0.001** | -0.099 (0.016) | **<0.001** |
|  | WMH*time | 0.029 (0.016) | 0.093 | 0.03 (0.017) | 0.077 |
|  | **Model B:** |  |  |  |  |
|  | Score_LA/SES_*Aβ*time | 0.052 (0.016) | **0.001** | 0.041 (0.016) | **0.008** |
|  | **Model C:** |  |  |  |  |
|  | Score_LA/SES_*WMH*time | 0.014 (0.019) | 0.462 | 0.010 (0.021) | 0.644 |

**Table S6. Summary of linear mixed-effect models examining SES excluding the years of education variable, Aβ, and WMH volume as predictors of longitudinal cognitive decline.**

Score_SES_exYoE_ indicates that SES was derived from a latent variable that did not include the Years of Education indicator.

|  | **Term** | **estimate** | **std.error** | **p value** |
| --- | --- | --- | --- | --- |
| Language | Score_SES_exYoE_*WMH*time | 0.062 | 0.049 | 0.211 |
|  | Score_SES_exYoE_*Aβ*time | 0.02 | 0.042 | 0.632 |
| Processing Speed/Attention | Score_SES_exYoE_*WMH*time | 0.004 | 0.035 | 0.911 |
|  | Score_SES_exYoE_*Aβ*time | -0.023 | 0.031 | 0.459 |
| Executive Function | Score_SES_exYoE_*WMH*time | 0.097 | 0.05 | 0.051 |
|  | Score_SES_exYoE_*Aβ*time | 0.007 | 0.043 | 0.872 |
| Episodic Memory | Score_SES_exYoE_*WMH*time | -0.031 | 0.058 | 0.589 |
|  | Score_SES_exYoE_*Aβ*time | 0.093 | 0.045 | **0.04** |
